# Simultaneous determination of sarcosine and its related metabolites by gas chromatography-tandem mass spectrometry for prostate cancer diagnosis

**DOI:** 10.17179/excli2018-1352

**Published:** 2018-10-16

**Authors:** Vichanan Yamkamon, Pyone Pyone Yee, Sakda Yainoi, Warawan Eiamphungporn, Thummaruk Suksrichavalit

**Affiliations:** 1 Department of Clinical Microscopy, Faculty of Medical Technology, Mahidol University, Bangkok 10700, Thailand; 2 Center of Data Mining and Biomedical Informatics, Faculty of Medical Technology, Mahidol University, Bangkok 10700, Thailand; 3 Department of Clinical Microbiology and Applied Technology, Faculty of Medical Technology, Mahidol University, Bangkok 10700, Thailand; 4 Department of Clinical Chemistry, Faculty of Medical Technology, Mahidol University, Bangkok 10700, Thailand

**Keywords:** gas chromatography-tandem mass spectrometry, sarcosine, alanine, glycine, creatinine, MRM

## Abstract

Shortly after sarcosine was delineated as a potential biomarker for prostate cancer in 2009, a variety of analytical methods for clinical application were developed. Moreover, higher uptake of glycine in the mitochondria also played a role in cancer proliferation. A major constraint in the accurate quantification of sarcosine was the interference of the two isomers, α-alanine and β-alanine, using chromatographic separation techniques. Accordingly, we aimed to develop an analytical method for determining sarcosine and its related metabolites (α- and β-alanine, glycine and creatinine) under the same conditions by gas chromatography-tandem mass spectrometry (GC-MS/MS). BSTFA + 1 % TMCS was used for silylation, and GC-MS/MS conditions were optimized for the target analytes. The unique transition ions of sarcosine, α- and β-alanine, glycine and creatinine set up in MRM acquisition were m/z 116 → 73, 190 → 147, 176 → 147, 176 → 147 and 100 → 73, respectively. This newly developed method was successfully validated to apply in clinical settings with low limits of detection (0.01 - 0.03 µg•mL-1), high correlations (R2 > 0.99), great accuracy (88 - 110 % recovery), and notable precision (RSD < 10 %). All TMS derivatives were > 80 % stable for up to 2 h after derivatization and analyzing during this period promises to achieve an accurate result. Monitoring the five-substance profile could enhance prospects for early diagnosis of prostate cancer.

## Introduction

The potential role of sarcosine (*N*-methyl derivative of glycine) as a potential prostate cancer biomarker was described by Sreekumar et al. in an unbiased metabolic profiling of prostate cancer, benign prostatic hyperplasia and healthy subjects (Sreekumar et al., 2009[30]). Elevated levels of sarcosine are strongly associated with prostate cancer progression and aggressiveness (Khan et al., 2013[19]). Sarcosine is an intermediate product of glycine's synthesis and degradation pathways. Two major enzymes, glycine *N*-methyl-transferase (GNMT) and sarcosine dehydrogenase (SARDH), regulate the biosynthesis and degradation of sarcosine, processes which involve folate metabolism and DNA methylation. It is noteworthy that exogenous exposure to sarcosine and glycine increases either cell invasion in benign prostate epithelial cells (Sreekumar et al., 2009[30]) or increases cell migration in metastasis of human prostate cancer cells (PC-3) (Heger et al., 2016[13]). Sarcosine also had considerable impact on overexpression of genes that involve in cell proliferation and cell cycle progression (Heger et al., 2016[14]). Moreover, metabolic foot printing of differential metabolites in NCI-60 cancer cell lines delineated the association of glycine uptake with cancer proliferation (Jain et al., 2012[15]). Accordingly, determination of sarcosine in parallel with glycine may be helpful in better understanding prostate cancer progression.

**Figure 1:**
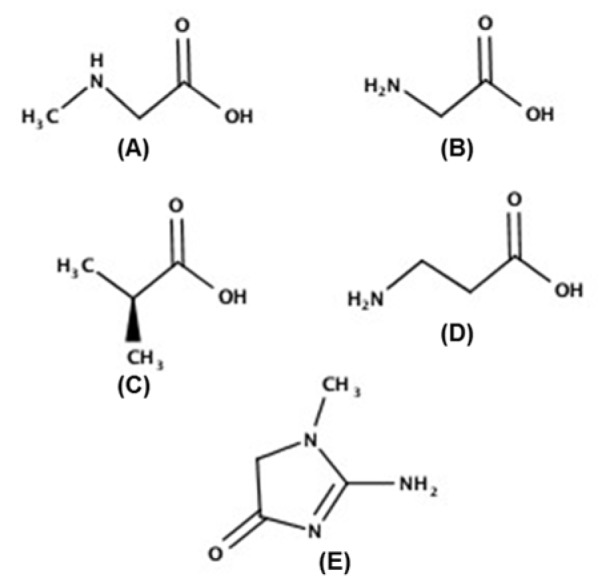
Chemical structures of sarcosine (A), glycine (B), α-alanine (C), β-alanine (D), and creatinine (E)

Analytical techniques for determination of substantial oncometabolite sarcosine in urine have been developed by colorimetric (Burton et al., 2014[3]; Yamkamon et al., 2018[34]), flow injection analysis with electrochemical detection (FIA-ED) (Cernei et al., 2012[6]), and chromatographic separation with mass spectrometric determination such as isotopic dilution gas chromatography mass spectrometry (ID-GC-M0S) (Wu et al., 2011[33]), capillary electrophoresis-tandem mass spectrometry (CE-MS/MS) (Soliman et al., 2012[29]), and high through-put high performance liquid chromatography-mass spectrometry (HPLC-MS) (Meyer et al., 2011[22]). Amongst them, the methods based on gas chromatography (GC) or liquid chromatography (LC) hyphenated, with mass spectrometry (MS) are popular owing to their high sensitivity and specificity. Despite this popularity, some studies failed to find an association between sarcosine level and prostate cancer progression (Jentzmik et al., 2010[17], 2011[16]). Thus, sarcosine as a prostate cancer biomarker is still controversial. Possibly influencing of analytical method on the interpretation of sarcosine is the presence of two alanine isomers, α- and β-alanine. They possess the same molecular weight (89.094 g•moL^-1^) and similar physiochemical properties (such as one active carboxylic group, amino groups and a side-chain methyl group) as shown in Figure 1(Fig. 1). Generally, higher amounts of α-alanine exist either exogenously in food or endogenously in the body than sarcosine and β-alanine. Therefore, accurate quantification of urinary sarcosine is a challenge. Liquid-liquid extraction with GC-MS using DB5ms columns (Shamsipur et al., 2013[26]) and LC-MS without derivatization (Meyer et al., 2011[22]) both have trouble separating sarcosine from the two alanine isomers. Accordingly, co-determination of sarcosine, glycine, and both α- and β-alanine has never been reported. Such analyses might benefit both diagnosis and the assessment of progression of prostate cancer. 

In the biomonitoring of urinary metabolites, concentrations of endogenous and exogenous metabolites and urine volume vary with water intake, physical activity and pathological conditions. In contrast, creatinine concentration depends only on muscle mass and has a relatively constant excretion rate, approximately 25 mg•kg^-1^•day^-1^. Urinary creatinine has been widely used to adjust analytical determinations of sarcosine to rectify possible dilution or concentrated effects (Heger et al., 2014[12]; Chen et al., 2014[7]). By using the enzymatic reaction based on the Jaffé reaction or automation, the sarcosine-to-creatinine ratio is calculated. A recent report showed that the urinary sarcosine/creatinine ratio can be used as a diagnostic indicator of prostate cancer (Weng et al., 2018[32]). If creatinine could be used to similarly adjust our targets of interest, time saving and reducing separate procedure for creatinine determination would be of benefit.

Therefore, development of a simple and accurate analytical method to determine sarcosine and related metabolites (glycine, α-alanine, β-alanine and creatinine) was deemed important to improve the diagnostics used for prostate cancer. This study first aimed to optimize a single condition for derivatization of all target substances which was suitable for GC-MS/MS. Second, the study sought to develop a state-of-the-art GC-MS/MS method for simultaneous determination of sarcosine and other relative metabolites in the synthetic urine for clinical applications. Monitoring of these five metabolites might favor early diagnosis of prostate cancer and a better understanding of its progression. 

## Materials and Methods

### Chemicals and reagents

Standards of sarcosine 98 %, α-alanine ≥ 99 % and β-alanine (BioUltra) ≥ 99.0 % (NT), glycine ≥ 99 % (HPLC), creatinine anhydrous > 98 % purity and ammonium chloride 99.5 %, A.C.S. were purchased from Sigma-Aldrich Corporation (Sigma-Aldrich, Saint Louis, MO, USA). *N, O*-Bis-(trimethylsilyl) trifluoroacetamide with 1 % trimethylchlorosilane [BATFA + TMCS, 99:1 (Sylon BFT)], derivatization grade for GC, was obtained from SUPELCO (Bellefonte, USA). Acetonitrile and water for chromatography grade were procured from Merck (Darmstadt, Germany). Urea 99.5 % AR and potassium phosphate dibasic AR were purchased from Loba Chemie Pvt. Ltd., (Mombai, India). Potassium chloride, calcium chloride dehydrate, sodium sulfate anhydrous, and sodium chloride were purchased from Merck.

### Standard preparation

Standard solutions (10 mL of 10 mg•mL^-1^ concentration) of sarcosine, α-alanine, β-alanine, glycine and creatinine were prepared as stock solutions. In brief, 0.1 g powder of each substance was completely dissolved in 10 mL water. Working solutions (1 mg•mL^-1^ concentration) were formulated with 2 mL stock standard solutions and 18 mL water, then separated as 1 mL individually into 20 aliquots of 1.5 mL polypropylene microcentrifuge tubes and stored at -20 °C until use.

### Synthetic urine preparation

Synthetic urine was used in this study for the purpose of urinary metabolite determination, including sarcosine, α-alanine, β-alanine, glycine and creatinine, replicating a clinical setting. It was prepared in the following concentrations: 25 g•L^-1^ urea, 1.60 g•L^-1^ potassium chloride (KCl), 1.103 g•L^-1^ calcium chloride (CaCl_2_), 2.25 g•L^-1^ sodium sulfate (Na_2_SO_4_), 2.295 g•L^-1 ^sodium chloride (NaCl), 1.4 g•L^-1^ potassium dihydrogen phosphate (KH_2_PO_4_) and 1.0 g•L^-1^ ammonium chloride (NH_4_Cl) (Rebelo et al., 2014[24]). However, creatinine was omitted from the synthetic urine because we would like to analyze creatinine in the urine (Ferenczy et al., 2016[11]). Synthetic urine was kept in the refrigerator at 2-8 °C until use.

### Derivatization (Silylation)

Standard solutions of 100 µL sarcosine and other metabolites were put into GC analyzed vials which were undergone overnight vacuum drying at 400 mmHg. After completely dry, anhydrous residues were reconstituted with 100 µL of BSTFA + 1 % TMCS for derivatization. Various parameters can vitally affect the productivity of the derivatization reaction. Among them, the reaction time was optimized by using four different durations (0.5 h, 1 h, 1.5 h and 2 h) at a fixed temperature (100 °C). Derivatives of each metabolite were mixed with 100 µL of acetonitrile, and then transferred into GC glass inserts. Afterwards, 1 µL of the derivatives of sarcosine, α-alanine, β-alanine, glycine and creatinine was injected into the GC-MS/MS by Gerstel MPS autosampler. The reaction time that produced the greatest yield for the five substances as an integrated peak area was fixed for use in further investigations.

### GC-MS/MS analysis

Bruker 456 Gas Chromatography (GC), coupled with a Bruker Scion Triple Quadrupole Mass Spectrometer (Bruker Corporation) and a GERSTEL multipurpose sampler MPS for GC, was used for this entire study. The GC conditions were: column, Rxi ®-5Sil MS (30 m×0.25 mm×0.25 µm, RESTEK, USA); carrier gas, helium; flow rate, 1.0 mL•min^-1^; splitless injection; injection port temperature, 260 °C; injection volume, 1 µL. Since nature of the analyte and column oven temperature have influence upon separation of target analytes, a gradient column oven temperature program was utilized for determining the complex substances. The column oven temperature program was as follows: 80 °C was initially maintained for 2 min and then raised to 280 °C at 15 °C•min^-1^; the temperature was held at 280 °C for 3 min. 

Tandem mass spectrometry conditions used in this study were: ion source temperature, 230 °C; ionization mode, electron impact (EI), ionization energy, 70 eV; collision-induced dissociation (CID) gas, argon; scan type, full-scan (FS) mode (mass range 50-500 a.m.u); selected ion monitoring (SIM) (Table 1(Tab. 1)) and multiple reaction monitoring (MRM). Empirically derived MRM was used instead of the built-in auto-optimization feature to identify the exact mass of the [M + H]^+^ ions in the spectrum. This was carefully figured out for particular precursor and product ions, and so optimized the collision energy (CE) for specific transition ions (Table 2(Tab. 2)). The ultimate MRM method for sarcosine, α-alanine and β-alanine, glycine and creatinine is shown in Table 3.(Tab. 3) The total run time was about 11 min.

### Method Validations

The processes to validate a newly developed method to be analytically acceptable were manipulated systematically under optimal experimental conditions (FDA, 2001[10]). Standard calibration plots were constructed, with the concentration ranges of 0.012 - 200 µg•mL^-1 ^for sarcosine and creatinine, and 0.03 - 500 µg•mL^-1^ for α-alanine, β-alanine and glycine, by using two-fold serial dilutions (n = 3). The linearity range of the linear equation was evaluated; the limit of detection (LOD) and the limit of quantification (LOQ) were interpreted when signal-to-noise ratios reached 3:1 and 10:1, respectively. In order to know the feasibility and reliability of this developed method for clinical application, our method was applied to synthetic urine. Three concentrations of spiked synthetic urine samples (1, 5 and 10 µg•mL^-1^) were analyzed (n = 20) to evaluate the recovery performance. It was calculated as Bianchi et al.:


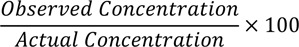


Mean percent recovery in the developed method should be within 80-120 of the target concentration to be accurate enough to use in routine laboratories (Bianchi et al., 2011[2]). 

To study random error and repeatability of the developed method, inter-day and intra-day precision studies were conducted. Twenty replicates of spiked urine samples with two different concentrations (1 and 5 µg•mL^-1^) were analyzed within one day and on four consecutive days with the developed GC-MS/MS method.

### Statistical analysis

The data required for interpretation of analytical method's validation were presented as mean ± SD, relative standard deviation (RSD, %), and regression analysis by using the Excel statistical software package (Microsoft Excel, 2016 version).

## Results and Discussion

### Optimal condition of silylation

**Figure 2:**
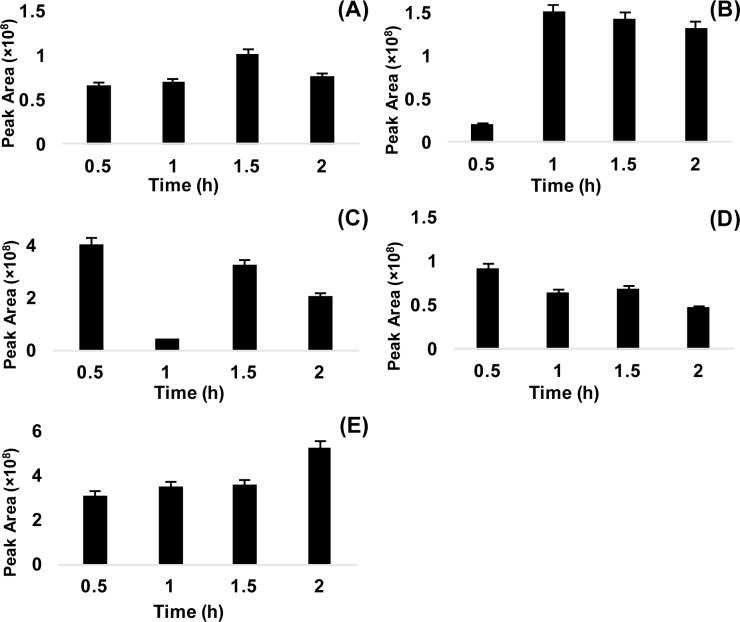
Optimization of silyl derivatization for five substances at various reaction times: sarcosine (A), α-alanine (B), β-alanine (C), glycine (D), and creatinine (E)

Derivatization was conducted to improve chromatographic separation and to improve suitability of sarcosine, α-alanine, β-alanine, glycine, and creatinine for GC-MS/MS analysis. The method of choice for derivatization was silylation, replacing low polarity functional group (trimethylsilyl) at the position of active hydrogen in the target substances (Orata, 2012[23]). Various derivatization methods have been utilized for amino acid analysis (Chen et al., 2014[8]; Zhu et al., 2016[35]). Silylation, alkylation and acylation are three major types of derivation reactions. Silylation is the most frequently used method due to its shorter reaction time, and greater volatility and stability. Sometimes alkylation/esterifcation needs a prolonged reaction time or a two-step procedure to complete derivatization. Even in silylation, common reagents include BSTFA [*N*,*O*-bis(trimethyl-silyl) trifluoroacetamide] and MTBSTFA [*N*-methyl-*N*-(tert-butyldimethylsilyl) trifluoroacetamide] (Little, 1999[21]). Derivatives from MTBSTFA are more stable than those of BSTFA which are moisture sensitive (Sobolevsky et al., 2003[28]), but no significant difference in derivatives yielded from small molecular mass compounds (Schummer et al., 2009[25]). Moreover, BSTFA's derivatives were more volatile as compared to those of MTBSTFA. Furthermore, the functional amino/imino groups of creatinine could be reacted with either MSTFA or MTBSTFA to convert trimethylsilyl or *tert*-butyldimethylsilyl derivatives (Siekmann, 1985[27]; Carobene et al., 1997[4]). Accordingly, the derivatization reagent BSTFA, in the presence of catalyst [1 % TMCS (trimethylchlorosilane)], was the best choice for this study. The procedure was adapted from a previous report (Wu et al., 2011[33]). To optimize the conditions, derivatization was carried out with different reaction times (0.5, 1, 1.5 and 2 h) at 100 °C. Each compound had distinct derivatization efficiencies with the four reaction times. The optimal reaction conditions were carefully evaluated, assessing reaction time vs relative peak area. The results showed that the reaction time of 0.5 h yielded the highest peak area for β-alanine and glycine, 1 h for α-alanine, 1.5 h for sarcosine, and 2 h for creatinine (Figure 2A-E(Fig. 2)). However, a reaction time of 1.5 h produced an alternative optimal yield for all substances with the second highest peak area at 100 °C, which was suitable for the five substances with GC-MS/MS.

### Compounds identification in full-scan monitoring

**Figure 3:**
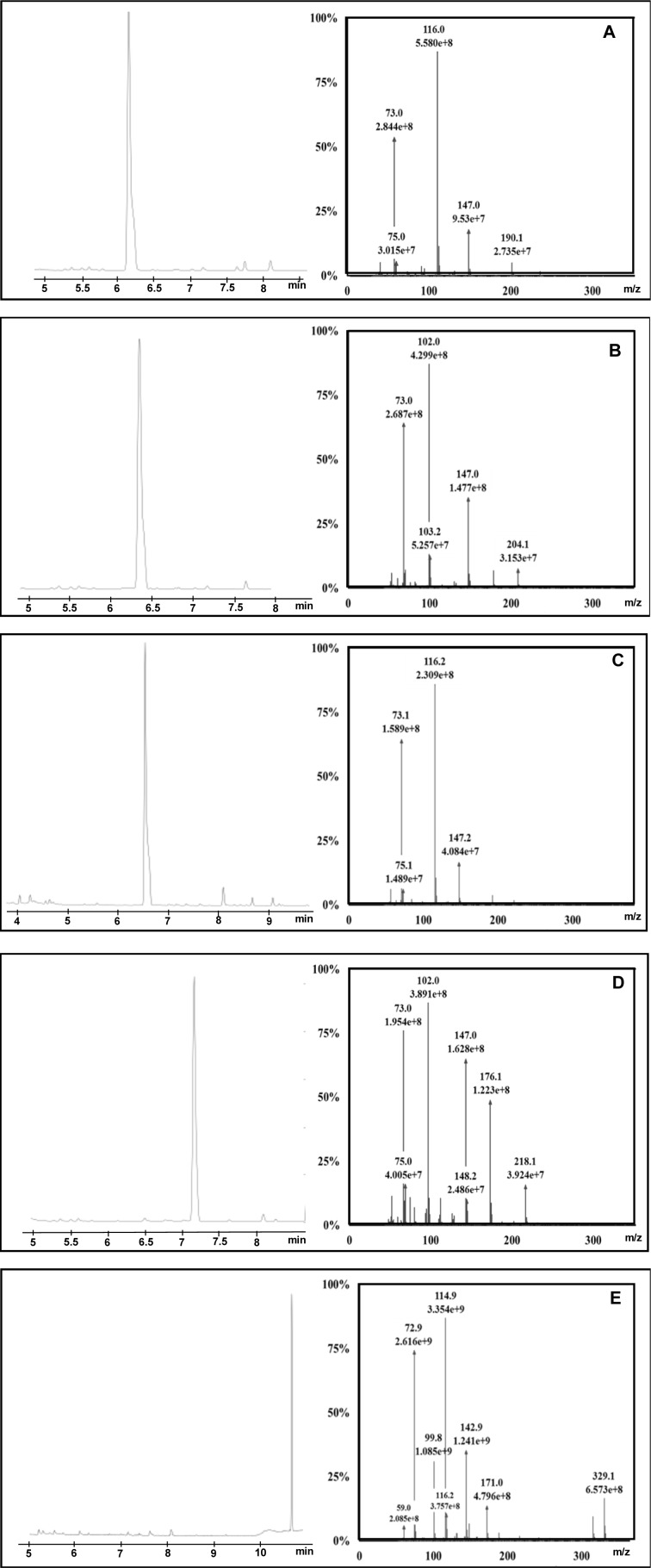
Chromatogram and mass spectrum of α-alanine (A), glycine (B), sarcosine (C), β-alanine (D), creatinine (E) in full-scan monitoring

Fundamental to GC-MS quantification are the mass spectrum and retention time (RT) of the target analyte. One µL derivative from 50, 100 and 200 µg•mL^-1^ concentrations of each substance was analyzed with full-scan (FS) monitoring by gas chromatography-tandem mass spectrometry (GC-MS/MS). All substances were readily eluted from the column with clear chromatographic separation under the designated temperature program. Herein, α-alanine was eluted first from the column at 6.14 min, while glycine, sarcosine, β-alanine and creatinine were sequentially eluted at 6.34 min, 6.53 min, 7.16 min and 10.68 min, respectively. Creatinine had the longest retention time among five; perhaps it had a stronger reaction with the stationary phase than did the others. In additions, the ring in creatinine might slow its volatility. Definitive mass spectra of sarcosine, α-alanine, β-alanine, glycine and creatinine were successfully identified (Figure 3A-E(Fig. 3)). Moreover, the retention time of each substance was very informative for compound identification as well. 

Despite the fact that retention time and mass spectrum matching were useful for compound identification, library search was not perfect for TMS derivatives since they had heavier molecular weights than intact compounds with different mass spectral patterns. TMS derivatives of sarcosine, α-alanine and glycine matched with the mass spectra of 2TMS sarcosine, α-alanine and glycine in the NIST (National Institute of Standard and Technology) Library (Lee et al., 2002[20]). However, TMS derivatives of β-alanine and creatinine could not be found in the NIST Library. Fortunately, top fragment ions of β-alanine *(m/z* 73, 176 and 218) were similar to the mass spectrum of 2TMS β-alanine in a drug bank database (Lee et al., 2002[20]). The mass spectral pattern of creatinine's derivative in our study was consistent with the claim of Siekmann (1985[27]). In summary, our target compounds were definitely identified, and their mass spectral patterns were consistent with previous research. Notably, the high abundance ions of sarcosine and α-alanine were similar both owned *m/z *73, 116, 147 and 190 as shown in Table 1(Tab. 1). Likewise, β-alanine and glycine had *m/z* 73, 102, 147 and 176. Only creatinine had significant molecular ions such as 73, 100, 115 and 143. Based on this information, MS/MS transition was vital to improve specificity and sensitivity of sarcosine, α- and β-alanine, glycine, and creatinine in the analytical determinations.

### Multiple reaction monitoring (MRM) transition

A GC-MS/MS method for determination of five substances in urine was developed which aimed for application in routine laboratories. Biological samples had matrix effects in GC-MS analyses, both with full-scan (FS) or selected ion monitoring (SIM) types. MS/MS fragmentation was the best suited to reduce matrix effect of urine samples, and increases sensitivity and specificity. MRM transition was evaluated starting from SIM; it was favorably generated by selecting three to four of the most abundance ions found on FS monitoring as target ions in SIM mode (Table 1(Tab. 1)). SIM MS supported a higher sensitivity and less complex chromatogram. An average sensitivity improvement of 11- to 22-fold was found when the FS and SIM methods were compared for airborne volatile organic compounds (Jia et al., 2006[18]). As expected, shifting from FS to SIM enhanced sensitivity by reducing background noise, and improved specificity by removing different molecular mass ions which were not included in the pre-defined list. However, it was not affordable to eliminate all potential matrix interferences. Due to this, product scan monitoring was conducted in pursuing the MRM setting and precursor ions were the same as target ions in SIM. Prominent precursor and product ions were chosen as transition ions for each compound. During transition ion selection, common molecular ion *m/z *73 was omitted because it was not target specific and was found in all the target analytes. Moreover, it is the most abundant molecular ion of BSTFA according to NIST Library (Lee et al., 2002[20]). Thereafter, we moved additional low abundance ions out with caution before collision energy (CE) optimization (carried out at 5 - 25 eV). Particular CE energies produce a variable abundance of fragment ions in equivalent chromatographic peak areas. Some transition ions did not produce any significant peak in the CE optimization (Table 2(Tab. 2)). Finally, two unique transition ions for sarcosine, four unique ions for creatinine, and five unique transition ions for glycine, α- and β-alanine were introduced into method acquisition for compound identification at specific retention times (Table 3(Tab. 3)). The most (100 %) abundant transition ions of sarcosine, α- and β-alanine, glycine and creatinine (*m/z *116 → 73, *m/z *190 → 147, *m/z *176 → 147, *m/z *176 → 147 and *m/z *100 → 73) were selected for quantitative analysis. All the rest were set up as qualifier transition ions.

**Figure 4:**
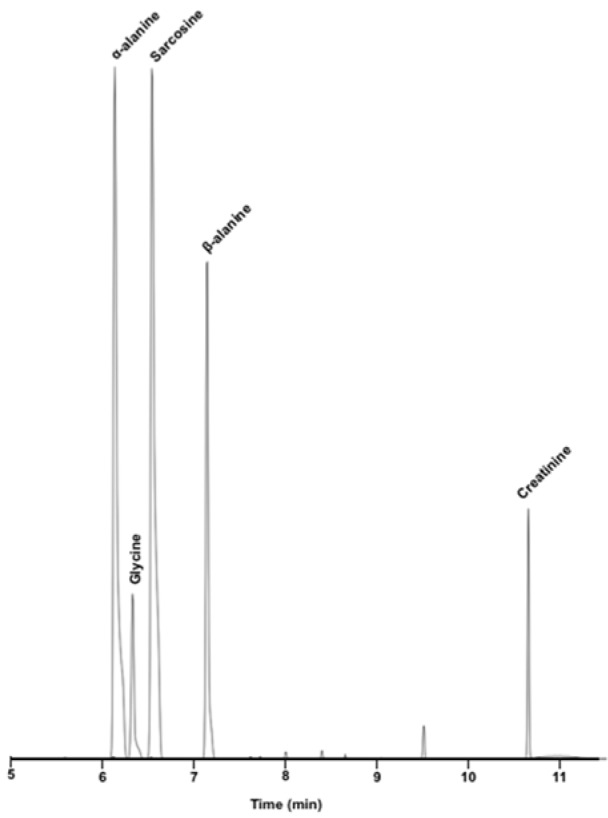
Total ion chromatogram (TIC) of five substances with Multiple Reaction Monitoring by GC-MS/MS: α-alanine (6.14 min), Glycine (6.34 min), Sarcosine (6.53 min), β-alanine (7.16 min), Creatinine (10.68 min)

Afterwards, 1 µL of 100 µg•mL^-1^ concentrations of five standard mixture was prosperously investigated in multiple reaction monitoring under optimal experimental conditions. Newly developed GC-MS/MS method was favorably separated as shown in Figure 4(Fig. 4) and significantly reduced background noise compared to full-scan mode. Accordingly, our research study decisively developed a useful GC-MS/MS method for simultaneous determination of urinary sarcosine, α-alanine, β-alanine, glycine and creatinine for prostate cancer detection. A number of advantages in our study was to begin with rapid GC-MS/MS run time, 11 min for all substances including holding time and requirement of single condition for all substances including sarcosine. Moreover, our method also has many benefits, such as simple derivatization procedure, shorter reaction times, readily available reagent, no additional protocol for creatinine analysis and regardless of time consuming. 

### Method validations and clinical application

In order to prove that the developed analytical method was acceptable for use in the clinical setting, method validations were performed. Fifteen points' calibration curves for sarcosine, α- and β-alanine, glycine and creatinine were constructed by doing two fold serial dilutions (Supplementary data). In Table 4(Tab. 4), high correlations of determination (*R*^2^ > 0.99) were observed for all five substances. The linearity of sarcosine was in the concentration range of 0.01-50 µg•mL^-1^ (*R*^2^ = 0.9954). Next, α-alanine and glycine were linear in the range of 0.03-500 µg•mL^-1^ with *R*^2^ values of 0.9974 and 0.9948, respectively. The linearity of β-alanine was in the range of 0.06-63 µg•mL^-1^ with *R*^2^ = 0.9926. Last, creatinine had an *R*^2^ 0.9923 in the linear range of 0.01-100 µg•mL^-1^. The limit of detection (LOD), and the limit of quantification (LOQ) were evaluated when signal-to-noise ratio (S/N) reached 3:1 and 10:1, respectively. The LOD and LOQ of sarcosine and creatinine were all ~0.01 µg•mL^-1^. The LOD and LOQ of α-alanine and glycine were each ~0.03 µg•mL^-1^, and for β-alanine were 0.03 and 0.06 µg•mL^-1^, respectively. This new method showed a low LOD for all substances.

According to Tan and Gajra (2006[31]) urinary α-alanine and glycine normal ranges in Caucasians are 191-531 µmol•L^-1^ and 142-297 µmol•L^-1^, however β-alanine is undetectable. The LOD and LOQ of α-alanine and glycine in our method were 0.03 µg•mL^-1^ (0.34 µmol•L^-1^ for α-alanine and 0.40 µmol•L^-1^ for glycine). Urinary metabolite concentrations can vary with dietary intake; anyhow, our method is sensitive enough to determine α-alanine and glycine routinely. In terms of sarcosine, Meyer *et al. *studied urinary sarcosine in men and women from China and report that the minimum sarcosine level is 25 ng•mL^-1 ^in men and 34 ng•mL^-1^ in women (Meyer et al., 2011[22]). Therefore, the LOD and LOQ of 0.01 µg•mL^-1^ (10 ng•mL^-1^) should be suitable to use in routine laboratories for sarcosine determination. The World Health Organization (WHO) recommends to recollect voided urine when creatinine < 30 mg•dL^-1^ (Barr et al., 2005[1]). Hence, the 0.01 µg•mL^-1^ (0.001 mg•dL^-1^) sensitivity of our method was sufficient. Thus, the high linearity and low LOD/LOQ of our developed method was sensitive and suitable for clinical application. 

With the purpose of analyzing urinary metabolites, our accuracy and precision studies utilized spiked synthetic urine. In terms of accuracy, mean recovery of known amounts of the five substances (1, 5 and 10 µg•mL^-1^) spiked into synthetic urine was 88-110 % (Table 5(Tab. 5)). This finding verified the accuracy of the GC-MS/MS methods, with satisfactory recoveries of the five urinary substances. To assess the precision of the developed method, the first step was to examine instrument precision or injection repeatability. Spiked synthetic urines with five standard mixtures were processed under optimal conditions of sample preparation. Two concentrations (1 and 5 µg•mL^-1^) of the spiked samples were assayed three times a day and run for twenty consecutive days. The second step was intra-assay precision. For that, twenty replicates of the spiked samples (1 and 5 µg•mL^-1^) were analyzed in a single run on a single day. The intra- and inter-run precisions were expressed as a percentage of relative standard deviation (RSD, %). The intra- and inter-run precisions of sarcosine were numerically below 5 % and 7 %, respectively (Table 6(Tab. 6)). This indicated a valid repeatability under optimal conditions. In the case of glycine and creatinine, both also achieved good intra- and inter-run precision with RSD < 10 %. In contrast, the precisions (RSD) with α- and β-alanine showed weaker repeatability, with RSD values of > 10 %. However, this was acceptable given our interest to separate sarcosine from the two isomers. The strength of our developed method was clearly the separation of sarcosine from them, and values of α- and β-alanine were not essential to quantify in analytical applications for the diagnosis of prostate cancer.

### Stability study of TMS derivatives

**Figure 5:**
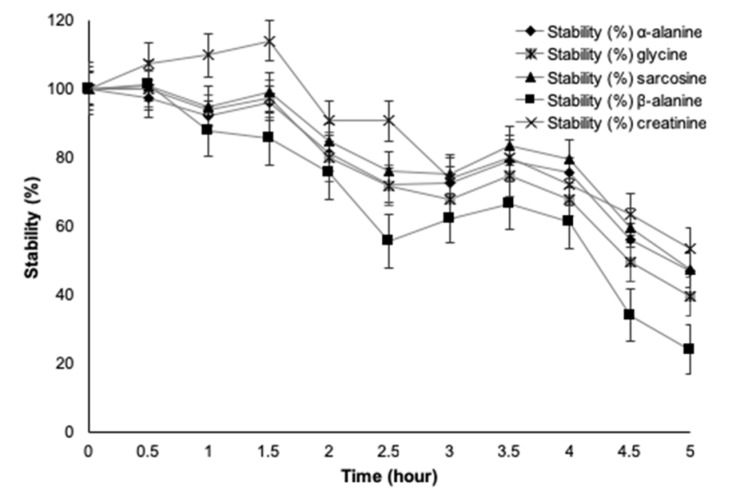
Stabilities of trimethylsilyl derivatives of five substances

Since the physiochemical properties of compounds may degrade, particularly concentration during the time of sample preparation, storage and analysis, stability of the assay was important to ensure a maximum allowable period for analyses. Spiked urine samples of sarcosine are stable at room temperature for 12 hours and also through three freeze-thaw cycles (Soliman et al., 2012[29]). In comparison, with storage conditions of -20 °C and -80 °C for 0-72 h, -80 °C appears more deleterious on sarcosine determination after 48 h of storage (Cernei et al., 2015[5]). It was interesting to know the stability of TMS derivatives of our target substances. Stabilities of TMS derivatives in terms of organic acids have been investigated (Christou et al., 2014[9]), however not for amino acids. With this purpose, a standard concentration (5 µg•mL^-1^) of five standard mixtures was investigated at thirty-minute intervals from time zero to five hours under optimal experimental conditions (Figure 5(Fig. 5)). We found that sarcosine, α-alanine, β-alanine, glycine and creatinine all had more than 80 % stability for up to two hours. Be aware of that investigation later than two hours after derivatization led to significantly diminished concentrations of target analytes. Determination of the five urinary substances within two hours after derivatization using gas chromatography-tandem mass spectrometry (GC-MS/MS) provided accurate quantification and the best results for clinical applications.

## Conclusion

A state-of-the-art method for determination of the prostate cancer potential biomarker, sarcosine, and its related metabolites including α-alanine, β-alanine, glycine and creatinine, was promising. Optimal conditions for silyl derivatization were 1.5 h at 100 °C. Unique transition ions for sarcosine (*m/z *116 → 73), α-alanine (*m/z *190 → 147), β-alanine and glycine (*m/z *176 → 147), and creatinine (*m/z *100 → 73) were successfully defined in MRM quantification. Progress in analytical method validation was considerable, demonstrating low limits of detection (0.01 - 0.03 µg•mL^-1^), with high correlation (*R*^2^ > 0.99), great accuracy (88-110 % recovery performance), and allowable precision (RSD < 10 %). Our studies showed stability of up to 80 % within two hours for all substances, and that it is better to analyse immediately or within 2 h of derivatization. This simple, robust and rapid GC-MS/MS analytic method is suitable to use in clinical applications. Monitoring a urinary profile containing five substances could provide a beneficial role in the early diagnosis of prostate cancer and a better understanding of its progression. 

## Acknowledgements

The authors acknowledge financial support from Health System Research Institute (HSRI: Grant No. 60-026) and National Research Council of Thailand (NRCT).

## Conflict of interest

The authors declare that there are no conflicts of interest related to this work.

## Supplementary Material

Supplementary data

## Figures and Tables

**Table 1:**
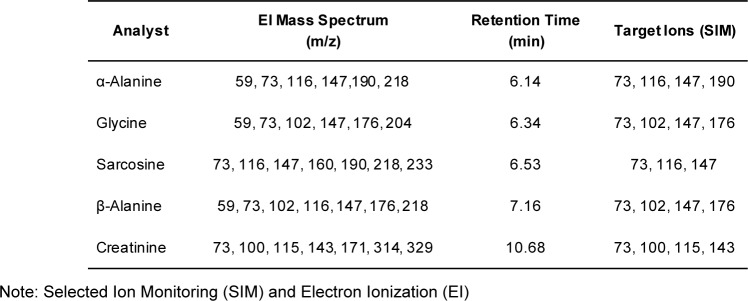
Summary of EI mass spectrum of five substances and target ions for SIM

**Table 2:**
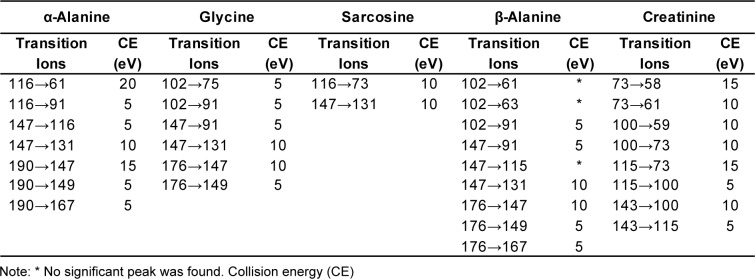
Collision energy optimization

**Table 3:**
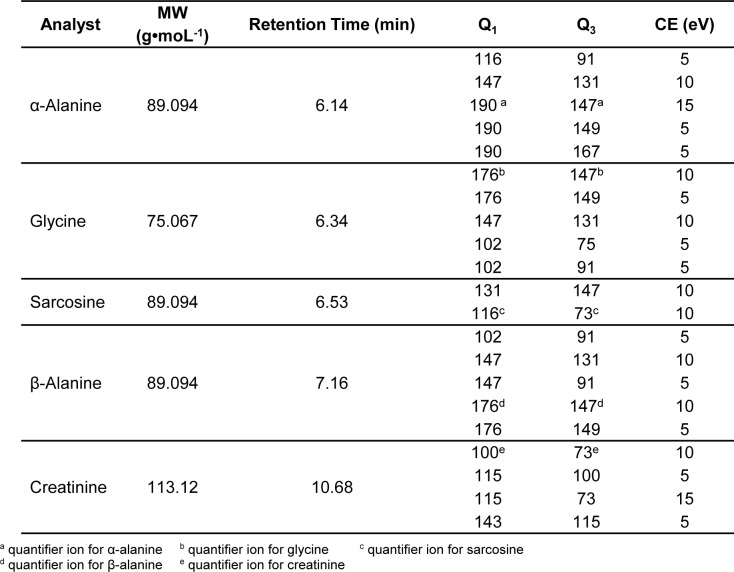
Summary of MS/MS parameters for five substances

**Table 4:**
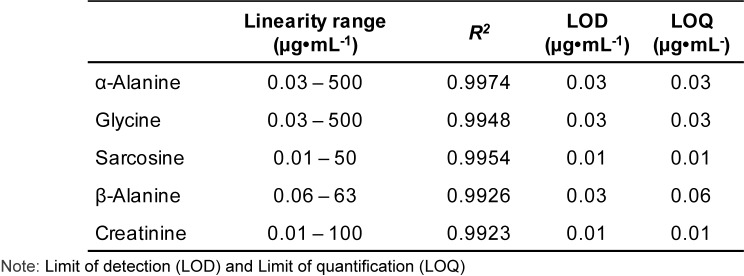
Linearity, LOD and LOQ of the five substances

**Table 5:**
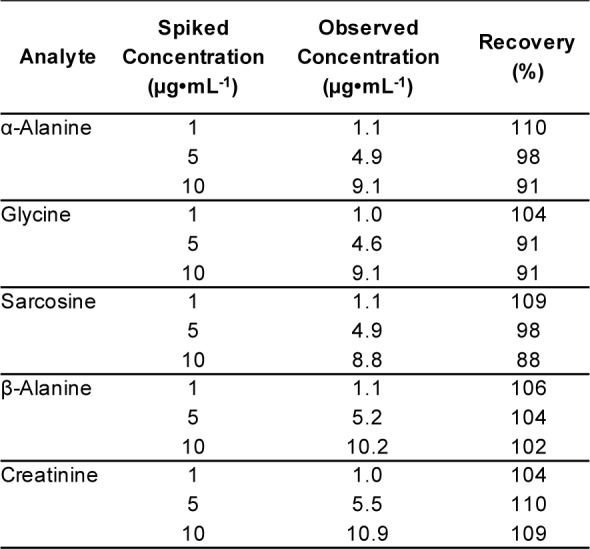
Recovery performance of spiked samples

**Table 6:**
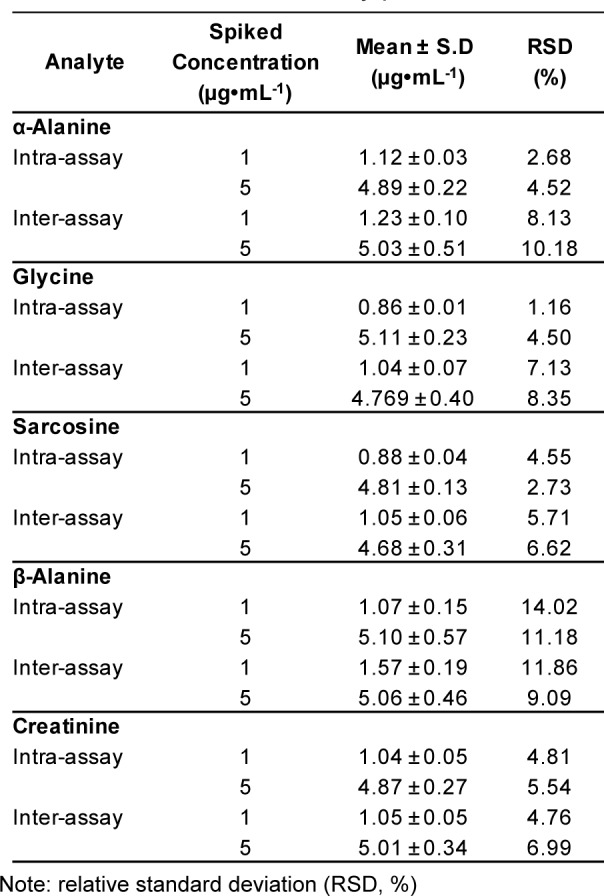
Intra- and inter-assay precision studies
